# Editorial

**DOI:** 10.2478/v10102-010-0017-z

**Published:** 2010-09

**Authors:** Mojmír Mach

**Affiliations:** Institute of Experimental Pharmacology & Toxicology, Dúbravská cesta Bratislava, Slovakia

COMMUNICATION – DIALOGUE – CO-OPERATION – SOLVING PROBLEMS

These are the words of nowadays Europe. Such approaches should be used in broad spectrum of life as well as in toxicology. It is therefore admirable that EUROTOX commissars, mainly past-president Prof. Liesivuori, invited all heads of European toxicological societies to discuss current issues of toxicology in Europe during IUTOX meeting held in Barcelona in July 2010. Renowned scientists and regulatory officers from Government, Industry and Academia made appearance and talked about contribution of toxicological research to society, importance of communication and education. The strategic meeting was successful. Prof. Liesivuori expressed gratitude to all delegates for the great suggestions and key findings resulting from the brainstorming sessions conducted during the meeting. Majority of delegates agreed that education in toxicology and related fields is the top priority for future. Communication towards society, in the sense of explaining possible risks, should be an important concern as well.

Taking a good example from EUROTOX, on the verge of summer the Slovak Toxicology Society SETOX organizes meeting of the toxicological societies from the Central Europe, i.e. Slovak, Czech, Polish, Austrian and Hungarian societies of toxicology. First positive step was done by Visegrad Fund and Federation of European Toxicologists & European Societies of Toxicology, which supported this meeting. It is essential for successful co-operation to establish strong connections, which can be further crucial for meaningful and highly profitable information to society.

I can only hope for success and wish for better and safer future

